# Transabdominal two-cavity approach for radical nephrectomy combined with inferior vena cava thrombectomy for malignant thrombus caused by renal cell carcinoma: a case series

**DOI:** 10.1186/s13256-018-1845-2

**Published:** 2018-10-25

**Authors:** R. Novotny, J. Chlupac, T. Marada, V. Borovicka, V. Vik, L. Voska, L. Janousek, Jiri Fronek

**Affiliations:** 1 Transplant Surgery Department, Institute for Clinical and Experimental Medicine, Videnska1958/9, 14021 Prague, Czech Republic; 2 First Faculty of Medicine, Charles University, Prague, Czech Republic; 3 Second Faculty of Medicine, Charles University, Prague, Czech Republic; 4 Department of Pathology, Institute for Clinical and Experimental Medicine, Prague, Czech Republic

**Keywords:** Renal cell carcinoma, Thrombus, Inferior vena cava, Thrombectomy, Nephrectomy, Transperitoneal

## Abstract

**Background:**

Advanced renal cell carcinoma in some cases causes malignant intravascular thrombus with the potential for growth into the inferior vena cava or even the right atrium. Renal cell carcinoma is accompanied by malignant intravascular thrombus in up to 10% of cases. We present an overview of three patients diagnosed as having renal cell carcinoma with malignant intravascular thrombus requiring radical nephrectomy combined with inferior vena cava thrombectomy.

**Case presentation:**

Three patients diagnosed as having renal cell carcinoma were indicated for renal cell carcinoma combined with inferior vena cava thrombectomy between 2014 and 2017 at our department: a 69-year-old white Caucasian woman, a 74-year-old white Caucasian woman, and a 58-year-old white Caucasian woman. According to the Novick classification of inferior vena cava tumor thrombus, there was one infrahepatic (level II) and two supradiaphragmatic (level IV) malignant intravascular thrombi. The average age of these patients was 67 years (range 58–74 years). All patients underwent radical nephrectomy combined with inferior vena cava thrombectomy through transabdominal approach. In patients with level IV malignant intravascular thrombus, transesophageal echocardiogram was used to guide the placement of the inferior vena cava cross-clamp above the diaphragm. In one patient the pericardium was opened to place a cross-clamp above a tumor just below the right atrium.

There were no postoperative mortalities to date with an average follow-up of 23 months (range 2–48 months). To date, no patient has demonstrated recurrent inferior vena cava malignant intravascular thrombus requiring secondary inferior vena cava thrombectomy or any other treatment. A comparison of estimated blood loss and transfusion rate was not significantly different in all three cases.

**Conclusion:**

Despite the technical complexity of the procedure, caval thrombectomy combined with radical nephrectomy currently represents the only radical treatment for renal cell carcinoma accompanied by malignant intravascular thrombus with good mid-term oncological outcomes.

## Background

Kidney cancer is the 14th most common cancer worldwide [1]. Renal cell carcinoma (RCC) accounts for 90 to 95% of malignant neoplasms arising from the kidney. The incidence of RCC differs greatly between regions, with the highest rates observed in the Czech Republic and North America [2]. Advanced RCC is in some cases accompanied by a malignant renal vein thrombus with potential to grow into the inferior vena cava (IVC) or even the right atrium (RA) [3]. RCC is accompanied by an intravascular malignant thrombus in up to 10% of cases [4]. This is considered to be a detrimental prognostic parameter reducing the patients’ 5-year cancer-specific survival by 17–36% [5, 6]. Surgical resection of the RCC combined with IVC thrombus removal has become the primary treatment option, with postoperative 5-year survival rate without metastasis ranging from 45–69% [7].

## Case presentation

### Patients

**Fig. 1:**
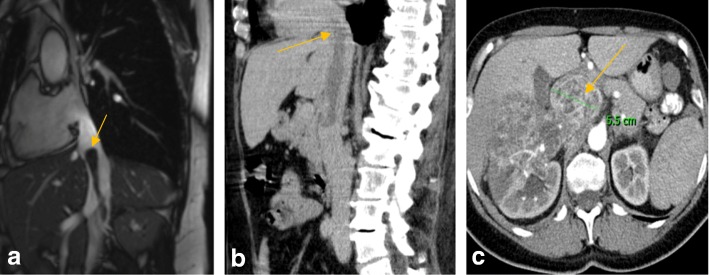
Preoperative computed tomography/magnetic resonance imaging of the renal carcinoma malignant tumorous thrombus level IV infiltration of the inferior vena cava (*arrow* marking the tumorous thrombus). **a** Patient 1: supradiaphragmatic tumorous thrombus without the right atrium infiltration. **b** Patient 2: suprahepatic tumor thrombus. **c** Patient 3: right kidney tumor with dilated inferior vena cava up to 5.5 cm

All patients were examined, diagnosed, and evaluated by a multidisciplinary team. Patients’ preoperative computed tomography and magnetic resonance images are summarized in Fig. 1. After a thorough discussion of the indications, and risks and benefits of the procedure, patients were approved for the procedure. All of the patients’ hospital stays are summarized in Table 1.

**Table 1:** Summary of patients’ characteristics

Patient number	Sex	Age	Renal functions before surgery		Preoperative CRP (mg/L)	Renal functions after surgery		Supradiaphragmatic access used	Pericardial resection	Inferior vena cava resection	Histology (Fuhrman classification)	Hostpital stay (days)	Follow-up (months)
			Urea (mmol/L)	Creatinine (μmol/L)		Urea (mmol/L)	Creatinine (μmol/L)						
1	Female	69	4.6	88.5	63.9	5.6	67.8	Subxiphoid access	No	No	Clear cell renal carcinoma Patient 1 - pT3b pNO pMO, grade 3/4	11	2
2	Female	74	4.8	98.7	35	10	129.7	Transdiaphragmatic	Yes	No	Clear cell renal carcinoma Patient 2 - pT3b pNO pMO, grade 4	8	18
3	Female	58	5	105.4	52.2	9.2	111.2	//////////////////////	No	Yes	Clear cell renal carcinoma Patient 3 - pT3a pNO, pMO, grade 3	6	48

*CRP* C-reactive protein

### Surgical procedure

All of the procedures were performed under full anesthesia. A midline laparotomy was performed in all of the patients without the differentiation of the left-sided or right-sided RCC. After the nephrectomy was performed, the IVC was dissected in a standard manner. The midline laparotomy allowed for an easy mobilization of the liver in patients where IVC cross-clamp had to be placed above the liver. In patients with a supradiaphragmatic malignant tumorous thrombus, a two-cavity (abdomen-thorax) procedure through a midline laparotomy was performed. The cross-clamp was placed above the diaphragm or just below the RA without the need for a sternotomy. We used either supradiaphragmatic or subxiphoid access for the cross-clamp placement. In these cases, a transesophageal echocardiogram (TEE) was used to monitor the positioning of the IVC cross-clamp to the thrombus localization.

### Patients follow-up

Every patient underwent an ultrasonographic check-up at 2-month and 6-month intervals after the procedure, followed by an annual ultrasonographic check-up.

### Results

#### Patient 1

A 69-year-old white Caucasian woman with right kidney tumor and IVC supradiaphragmatic thrombus with no relevant medical history was referred to our department for treatment. Based on computed tomography angiography (CTAG) the tumor was classified as level IV (Fig. 1). The results of a laboratory evaluation before the procedure were: hemoglobin (Hb) 92 g/L, C-reactive protein (CRP) 63.9 mg/L, urea 4.6 mmol/L, creatinine 88.5 μmol/L, and white blood cells (WBC) 10.2 × 10^9^/L.

**Fig. 2:**
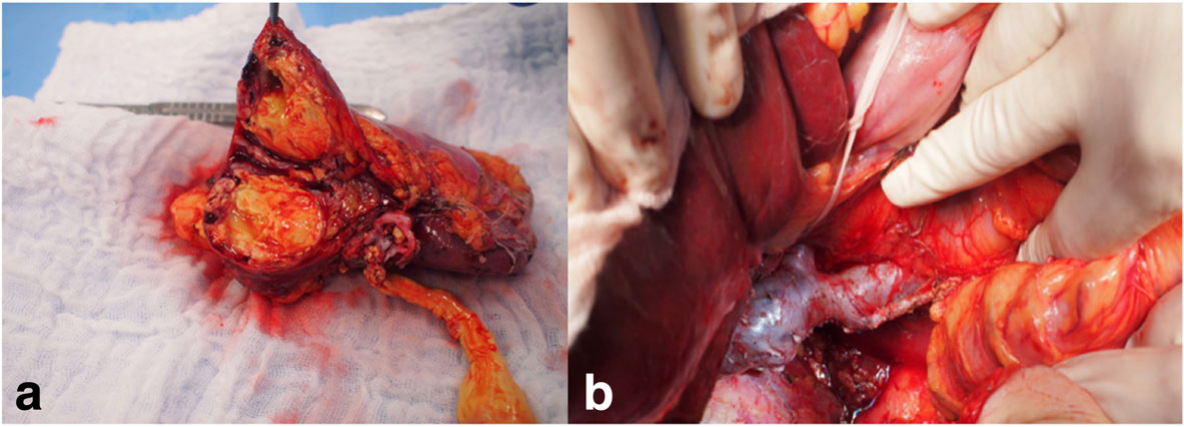
Periprocedural pictures. **a** Patient 1: right kidney with a tumor 40 × 37 × 35 mm and malignant thrombus in the renal vein. **b** Patient 3: residual 40% stenosis of the inferior vena cava after cavectomy closure with primary suture and radical nephrectomy

A right nephrectomy was performed in a standard manner with a tumor of the size 40 × 37 × 35mm (Fig. 2). TEE was used to determine the position of the IVC clamp with reference to the tumor position. Based on TEE, the pericardium was resected, and an IVC clamp was placed just below the RA through subxiphoid access. A cavotomy was performed with the extraction of tumor thrombus 85 × 35 mm. The cavotomy and laparotomy were closed in a standard manner using non-absorbable monofilament running suture.

**Fig. 3:**
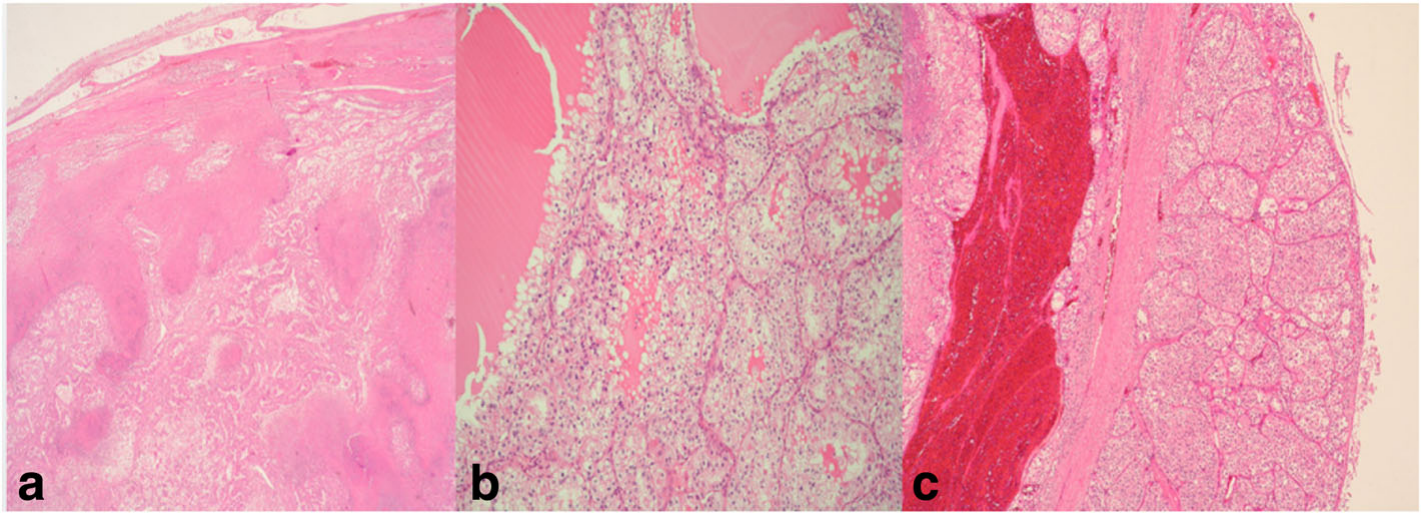
Clear cell renal carcinoma histology, grading 1–4 (Fuhrman classification). Hematoxylin and eosin stain. **a** Patient 1 - pT3b pNO pMO. **b** Patient 2 - pT3b pNO pMO. **c** Patient 3 - pT3a pNO, pMO

She was discharged on the 11th postoperative day with urea 5.6 mmol/L and creatinine 67.8 μmol/L. Her postoperative period was uneventful. Tumor histology revealed clear cell renal carcinoma Patient 1 - pT3b pNO pMO, grade 3/4 (Fuhrman classification) (Fig. 3). She is alive was without recurrence of RCC and/or IVC tumor thrombus at a 2-month follow-up.

#### Patient 2

A 74-year-old white Caucasian woman with left kidney tumor and supradiaphragmatic IVC malignant thrombus reaching the RA with no relevant medical history was referred to our department for treatment. Based on CTAG the tumor was classified as level IV (Fig. 1). The results of a laboratory evaluation before the procedure were: Hb 92 g/L, CRP 35 mg/L, urea 4.8 mmol/L, creatinine 98.7 μmol/L, and WBC 10.6 × 10^9^/L.

Her left kidney and IVC were dissected, liver mobilized. Atrial thrombus was confirmed using TEE. First, a nephrectomy of the left kidney was performed in a standard manner. The tumor size was 75 × 80 × 72mm. Based on the TEE the thrombus in the RA was hardly attached to its wall. A transdiaphragmatic approach was used. A circular suture was placed on the RA where the thrombus was attached, the RA was opened, and the thrombus was flushed out. The RA was closed with primary suture. TEE confirmed the removal of the entire thrombus; therefore, there was no need to remove the thrombus with the use of extracorporeal circulation. In the end, a cavotomy was performed, and the malignant thrombus was removed. The cavotomy closure was performed with primary suture.

On the 8th postoperative day, our patient was transferred to the department of internal medicine for further treatment with urea 10 mmol/L and creatinine 129.7 μmol/L. Tumor histology revealed clear cell renal carcinoma Patient 2 -  pT3b pNO pMO, grade 4 (Fuhrman classification) (Fig. 3). She is alive and was without recurrence of RCC and/or IVC tumor thrombus at an 18-month follow-up.

#### Patient 3

A 58-year-old white Caucasian woman with right kidney tumor and IVC thrombus with no relevant medical history was referred to our department for treatment. Based on CTAG the tumor was classified as level II (Fig. 1). The results of a laboratory evaluation before the procedure were: Hb 83 g/L, CRP 52.2 mg/L, urea 5 mmol/L, creatinine 105.4 μmol/L, and WBC 12.2 × 10^9^/L.

The right kidney and IVC were dissected in a standard manner. IVC was dilated up to 5 cm just under the right ventricle (RV). A right nephrectomy was performed in a standard manner. The tumor dimensions were 120 × 75 × 70 mm. An IVC cross-clamp was placed just under the liver. The tumorous thrombus was removed through cavotomy in two pieces (65 × 40 × 40 mm and 42 × 30 × 32 mm) as we were unable to remove it at one attempt due to IVC wall infiltration. The IVC wall was infiltrated with a tumor; therefore, it was also resected. The cavectomy was closed with a primary suture (residual stenosis after the closure of the cavectomy was around 40%) (Fig. 2).

She was discharged on the 6th postoperative day with urea 9.2 mmol/L and creatinine 111.2 μmol/L. Her postoperative period was uneventful. Tumor histology revealed clear cell renal carcinoma Patient 3 -  pT3a pNO, pMO, grade 3 (Fuhrman classification) (Fig. 3). She is alive and without recurrence of RCC and/or IVC tumor thrombus at a 48-month follow-up.

## Discussion

Advanced RCC with IVC thrombus has an unfavorable prognosis. Even in cases where no metastasis is revealed it is accompanied by cancer-specific death in 29% of cases [8, 9]. Surgical resection of IVC tumor thrombus prolongs a patient’s survival rate. A study by Tang *et al*. demonstrated that nephrectomy combined with IVC tumor thrombus resection had achieved a 62.9% survival in 5 years and 56% in 10 years [8].

The surgical strategy for IVC tumor thrombus resection depends on the level of the thrombus extension. The most complex surgical strategy is usually required for patients with level IV thrombus. These technically challenging procedures are accompanied by 30% morbidity and 15% mortality with frequently used extracorporeal circulation and hypothermic circulatory arrest in order to create optimal conditions for tumor extirpation [10].

RCC with IVC tumor thrombus is classified based on the thrombus level and its relationship to the hepatic venous system. This is important for the determination of the safest and most effective method for the surgical procedure. The first classification was introduced by Libertino in 1986, followed by Wilkinson *et al*. and Pritchett *et al*. [3, 11–13]. These classifications had a major drawback. They grouped intrahepatic and retrohepatic IVC thrombi in the same category despite the fact that their surgical approach greatly differs. To this day, there is no commonly used standardized classification.

When considering tumor reoccurrence or progression, there is a high insistence on determining a prognostic marker that would screen the patients in need for further monitoring or eventual oncologic treatment. The consistency of a thrombus impact on a cancer-specific survival in patients with RCC was extensively assessed by Weiss *et al*. and Antonelli *et al*. as a possible prognostic factor [14, 15]. The study published by Mager *et al*. confirmed that patients with RCC tumor thrombus level I–II showed higher survival rate compared to levels III–IV [4]. Also, the level of thrombus is an independent prognostic marker in patients without metastases [4, 16].

Our novel surgical approach allows us to perform radical nephrectomy combined with IVC thrombectomy especially in patients with supradiaphragmatic thrombus through a single incision, upper midline laparotomy. Our surgical strategy eliminates the need for full or partial sternotomy, and thoracotomy in patients with supradiaphragmatic thrombus where extracorporeal circulation is not needed. We combine either transdiaphragmatic or subxiphoidal access to place a supradiaphragmatic cross-clamp on the IVC under TEE guidance. TEE is an essential tool for safe IVC cross-clamp placement and thrombus extraction. Our approach greatly minimizes the invasiveness of the entire procedure and allows us to control and monitor the IVC thrombectomy in real time.

The quality of life in patients with cancer is greatly affected by thromboembolic complication. Patients with RCC and small residual tumor thrombus are almost seven times more likely to develop venous thrombosis (VTE), and eight times more likely to develop VTE than patients with RCC without tumorous thrombus [17]. A very limited amount of data is available for the determination of the optimal anticoagulation treatment or secondary prophylaxis in these patients. A study by Lee *et al*. demonstrated lower recurrent VTE among patients with dalteparin therapy over orally administered anticoagulation [18].

## Conclusions

Patients with advanced RCC have a low survival rate without surgical intervention. Despite the technical complexity of the procedure, caval thrombectomy combined with radical nephrectomy currently represents the only radical treatment of RCC accompanied by a malignant thrombus in the IVC. Our novel surgical approach through a midline laparotomy allows us to perform a transabdominal two-cavity approach for radical nephrectomy combined with IVC thrombectomy without the need of a sternotomy. Also, the use of TEE allows us to safely guide a cross-clamp above the malignant thrombus in patients with level IV malignant intravascular thrombus.
